# Associations of Agency and Communion With Domain-Specific Self-Perceptions of Aging: A Cross-Sectional Study In Old-Old Adults in Poor Health

**DOI:** 10.1177/00914150211050874

**Published:** 2021-10-18

**Authors:** Anne Blawert, Sarah K. Schäfer, Susanne Wurm

**Affiliations:** Department for Prevention Research and Social Medicine, Institute for Community Medicine, 60634 University Medicine Greifswald, Greifswald, Germany

**Keywords:** personality, agency, communion, views on aging, self-perceptions of aging, old age

## Abstract

A large body of research indicates that self-perceptions of aging (SPA) play an important role for health in later life. Hence, more research on SPA and correlates is needed, especially in old age and poor health, where negative SPA tend to prevail. Recent studies identified personality as an important correlate of SPA in young-old and relatively healthy samples. Thus, we investigated cross-sectional associations of agency and communion with two SPA domains in a sample of old-old adults in poor health (*n*  =  154; *M_age_*  =  81.55, *SD*  =  4.56, 58.4% women). In multiple regression analyses, agency and communion were associated with SPA related to ongoing development beyond health. In contrast, only health as a covariate was significantly associated with SPA related to physical losses. Thus, personality may be a resource associated with gain-related SPA, at least for those in poor health and old age.

In recent years, self-perceptions of aging (SPA), that is, what people think about their own age and aging, have emerged as an important concept in research on health in later life (for an overview, see e.g. Wurm et al., 2017). Thus, it is important to better understand which constructs are associated with SPA. Recent studies have suggested that SPA is related to personality (e.g., Kornadt et al., 2019, 2020; Rupprecht et al., 2019), which is defined as characteristic ways of thinking, feeling and behaving that distinguish people from one another (Kandler et al., 2014). However, these studies have focused almost exclusively on the Big Five personality traits and mostly considered young-old samples in relatively good health (e.g., O'Shea et al., 2017; Rupprecht et al., 2019). Yet, investigating correlates of SPA is especially important in situations where positive SPA are challenged and negative SPA are confirmed; this is particularly the case in old age and poor health, where people tend to associate their aging more strongly with physical losses and less with ongoing psychological development (Diehl et al., 2021; Wurm et al., 2020). To advance understanding of the relationship between personality and SPA in this setting, the present study explored associations of the “Big Two” meta-traits of personality, namely agency and communion (Martin & Slepian, 2020), with SPA in a sample of old-old adults in poor health.

## SPA in Old Age

The concept of SPA gained in interest in the 1970s with the development of the Attitudes Toward Own Aging Scale (ATOA; Lawton, 1975), a unidimensional measure ranging from negative to positive SPA. Since then, the number of studies on the role of SPA in later life has increased immensely (cf. Westerhof et al., 2014; Wurm et al., 2017), suggesting that SPA may constitute an indicator of health and wellbeing, adjustment to age-related changes and overall successful aging across the second half of life up to old age (Baltes & Smith, 2003; Kleinspehn-Ammerlahn et al., 2008; Levy, 2003).

More recent measures like the Attitudes to Aging Questionnaire (Laidlaw et al., 2007), the Expectations Regarding Aging Scale (Sarkisian et al., 2002) and the AgeCog Scale (Steverink et al., 2001; Wurm et al., 2007) differentially assess perceptions of age-related gains and losses, thus acknowledging the multidimensional and multidirectional nature of adult development (Baltes, 1987; Kornadt et al., 2019, 2020). Two domains that have been found to be relevant for health in later life are the SPA domains of physical losses (i.e., declining health and fitness) and ongoing development (i.e., expanding capabilities and pursuing plans) as assessed by the well-established Agecog Scale (Diehl et al., 2021; Westerhof et al., 2014; Wolff et al., 2014; Wurm et al., 2017).

With advancing age, individuals perceive their aging more strongly as associated with physical losses and less with ongoing development (Diehl et al., 2021). This change in subjective perceptions of aging reflects, in part, objective age-related changes: The likelihood of experiencing disease, physical losses and limitations in one's activities increases in old age (Baltes & Smith, 2003). Yet, older adults can simultaneously perceive their aging as associated with ongoing development. It is thus important to shed light on factors that help to explain interindividual differences in the way people perceive their own aging.

## Personality and SPA

Several studies point to associations of personality and SPA. A study on personality and subjective age, a construct related to SPA, found that this association seems to become stronger with age (Stephan et al., 2012), making the investigation of the relationship between personality and SPA in old age even more important. However, studies investigating personality and SPA have been mostly conducted in young-old, relatively healthy samples or large surveys of the general population. Such survey samples are known to be biased in terms of health, age, and socioeconomic status (Enzenbach et al., 2019).

A further conceptual limitation of previous studies on personality and SPA is that they primarily endorsed personality on the level of the Big Five traits. In these studies, neuroticism seems to be consistently associated with negative or loss-related SPA (e.g., Loi et al., 2015; Moor et al., 2006). Regarding the other traits, however, findings are inconsistent across studies and measures of SPA (e.g., Moor et al., 2006; Rupprecht et al., 2019). To add to the conceptual understanding of associations of personality and SPA, we thus examined the most basic level of personality, namely, the meta-traits of agency and communion. These two meta-traits emerge as higher-order factors of different personality levels such as the Big Five traits and personal values (e.g., Vecchione et al., 2011; for an overview see e.g., Blawert & Wurm, 2019).

Since the 1970s, agency and communion were first investigated as gender-related traits labeled masculinity and femininity (Bem, 1974; Spence et al., 1974), later as instrumental and expressive traits or agency and communion in order to overcome the strong focus on gender of earlier work on these traits. Agency (or masculine/instrumental traits) reflects a focus on the self and is associated with qualities such as dominance, decisiveness, and pursuing one's own goals. Communion (or feminine/expressive traits) reflects a focus on others and is associated with qualities such as warmth, the ability to build up and sustain positive social relationships, and helping others.

Despite their diverging focus, agency and communion are not mutually exclusive; a person can be highly agentic and at the same time highly communal, while another person might score low on both traits or high on one and low on the other. Agency and communion are inherently positive qualities and ideally, a person incorporates both agentic and communal traits, thus caring for the well-being of others without losing sight of personal needs (Bakan, 1966).

In early studies mainly conducted in young adults, this was expressed in the androgyny hypothesis suggesting that those high in both agency and communion (termed androgynous) could adapt most flexibly and successfully to situational demands (Bem & Lewis, 1975). However, some initial studies found agency (but not communion) to be associated with successful adaptation as reflected in mental health outcomes (masculinity model; for an overview, see Lefkowitz & Zeldow, 2006). In contrast, several more recent studies suggest that in later life, higher agency and communion are both associated with higher well-being (Helgeson, 1994; Matud et al., 2020) and life satisfaction (Dean-Church & Gilroy, 1993; Welzel & Inglehart, 2010). In addition, androgynous individuals report lower scores of depressive symptoms (Vafaei et al., 2016). High agency and communion may thus represent a prerequisite for successful aging (Perrig-Chiello & Hutchison, 2010). The integration of agency and communion is also considered a central component of generativity (Ackerman et al., 2000; Mansfield & McAdams, 1996; McAdams & de St Aubin, 1992), which might be perceived as a positive part of the aging experience. Moreover, the finding that agentic and communal themes in autobiographical memories foster optimism for the future (Austin & Costabile, 2017) might further translate into the perception of age-related ongoing development, given that optimism is associated with more positive SPA (Turner & Hooker, 2020).

Finally, agency is associated with positive self-evaluation (Abele & Hauke, 2020) and both agentic and communal themes emerge when older adults are asked to describe themselves freely (Diehl et al., 2004), pointing to the relevance of both meta-traits for the perception of the self (Paulhus & Trapnell, 2008). While, to our knowledge, associations of SPA with agentic and communal *traits* have never been examined, we found associations with agentic and communal *personal values* in an earlier study. Using population-based, longitudinal data of adults aged 40 + from Germany, that study demonstrated that agentic and communal personal values are differentially related to change in SPA domains (Blawert & Wurm, 2021): The growth-related agentic value of openness to change as well as the growth-related communal value of self-transcendence were found to predict increases in SPA related to ongoing development over the course of three years, while self-transcendence additionally predicted decreases in SPA related to social losses during the same period. These findings suggest domain-specific associations of agency and communion with SPA.

## The Present Study: Agency, Communion and SPA in Old-Old Adults in Poor Health

In this study, we considered domain-specific associations of agentic and communal traits with SPA related to ongoing development and SPA related to physical losses. Given the findings reviewed above, we expected agency and communion to be related to SPA ongoing development beyond health. Regarding SPA physical losses, we hypothesized that – due to its more explicit focus on physical decline – agency and communion are not associated with this SPA domain when controlling for health.

## Methods

### Participants and Procedure

Data came from the intervention study TIGER (Transsectoral Intervention Program to Improve Geriatric Care in Regensburg). This study took place in the city of Regensburg in Southern Germany. Two hundred and forty-four older adults were recruited in a hospital and randomized into intervention and control groups. Starting in hospital, participants of the intervention group were supported by specialized nurses to prevent readmission to hospital over the course of one year. Details of the intervention are presented in Rimmele et al. (2021). For the present analyses, we combined data from the intervention and control groups that was collected one month after discharge from hospital using self-report questionnaires.

Inclusion criteria for the study were a minimum age of 75, living in a radius of 50 km from the city of Regensburg, a Mini-Mental State Examination (MMSE) result of at least 22 points, insurance by the statutory health insurance AOK Bavaria, admission to the hospital Barmherzige Brüder in Regensburg, and discharge to their own homes.

Exclusion criteria were planned discharge to a nursing home, palliative care situation, and planned readmission to the hospital within the next four weeks.

### Measures

*Agency and communion* were measured with two subscales of the German Extended Personal Attributes Questionnaire (Runge et al., 1981). Participants were asked to rate how they would describe themselves in terms of eight agentic (e.g., independent, active) and eight communal (e.g., helpful, friendly) adjectives on a five-point Likert-scale (0  =  *not at all* to 4  =  *very much*). Item scores of the respective subscales were averaged and higher scores reflected higher agency or communion.

*Self-perceptions of aging* were measured with the AgeCog Scales (Steverink et al., 2001; Wurm et al., 2007). We used the two subscales of SPA ongoing development and SPA physical losses, each comprised of four items starting with the item stem “Aging means to me…”. An example item for SPA ongoing development is “Aging means to me that I continue to make plans”. An example item for SPA physical losses is “Aging means to me that I am less vital and fit”. Participants reported their agreement with each item on a four-point Likert-scale (0  =  *not at all* to 3  =  *very much*). Scores were averaged over the respective subscale. A higher score reflects higher agreement that aging is associated with ongoing development or physical losses.

*As covariates* we included emotional and physical health as assessed with the 12-item Short-Form Health Survey (Ware et al., 1996) to account for health status after a hospital stay and its known association with SPA. Item examples are “Does your health now limit you in climbing several flights of stairs?” (physical health; three-point Likert-scale; *yes, limited a lot, yes, limited a little* and *no, not limited at all*), and “How much of the time during the past four weeks did you have a lot of energy?” (emotional health; 7-point Likert-scale from *all of the time* to *none of the time*). Scores were coded so that a higher score indicates better health. All analyses were additionally controlled for age in years, sex (male or female), and education according to the International Standard Classification of Education (ISCED; UNESCO, 1997).

### Analytical Procedure

All analyses were conducted using *RStudio* (R Core Team, 2019) and the *lavaan* package (Rosseel, 2012). We conducted *t*-tests for independent samples to compare study variables between our study sample and participants who did not provide sufficient data to be included in our analyses as well as between treatment and control group. To investigate bivariate associations between agency, communion, SPA, physical and mental health, we conducted zero-order correlation analyses. To examine unique relationships between agency, communion, SPA and emotional and physical health, we conducted multiple regression analyses. Multicollinearity was assessed using Variance Inflation Factors (*VIF*), with *VIF* ≥ 10 indicating multicollinearity between predictors (O’brien, 2007). For missing data, we used multiple imputations based on the packages *mice* (van Buuren & Groothuis-Oudshoorn, 2011) and *miceadds* (Robitzsch & Grund, 2021) for *R.* Following recommendations (Van Buuren, 2018; Zhang, 2016), 20 datasets were imputed based on the study variables. Pooled results from these datasets are presented as results of the regression and correlation analyses. For post-hoc power analyses, G*Power version 3.1 (Faul et al., 2009) was used.

## Results

### Sample Characteristics

Participants were included in the analyses if they provided information on at least one of the variables of interest, that is, agency, communion, SPA ongoing development and SPA physical losses. We thus included data of 154 older adults (58.4% women) with a mean age of 81.67 years (*SD*  =  4.55) in our analysis. Respondents reported relatively low physical health (*M*  =  31.71, *SD*  =  9.86) compared with normative values from the German general population (Wirtz et al., 2018), *t*(110)  =  −8.94, *p* < .001, while not differing in emotional health (*M*  =  45.29, *SD*  =  11.69), *p*  =  .610. As expected for a sample of old-old adults in poor health, the dropout rate was relatively high in this study: Of the initially recruited 244 participants, 208 took part in the assessment one month after discharge from hospital and only 154 of these (63.11%) provided data for the cross-sectional statistical models of interest in this study. Of those n  =  36 participants who did not take part in the T1 assessment, three (8.3%) died, five (13.9%) moved into a nursing home and thus discontinued the study and two (5.6%) reported a momentary break from the study. The remaining 26 (72.2%) participants did not return questionnaires, thus also counting as non-participants in the T1 assessment. However, participants who dropped out did not differ from our study sample in age, *t*(242)  =  1.18, *p*  =  .241, sex, *χ^2^*(1)  =  0.19, *p*  =  .660, and educational level, *t*(188)  =  −0.15, *p*  =  .880. Moreover, the treatment and control groups did not differ in variables of interest, *p*  ≥ .071. Therefore, participants of both groups were included in our analyses.

### Multiple Imputations

Multiple imputations were used to handle missing data (see Table 1 for percentages of missing data per variable) to ensure sufficient statistical power (Zha & Harel, 2021). We assumed missing data to be completely at random, which was supported by a non-significant Little's MCAR Test, *χ^2^*(99)  =  108.00, *p*  =  .258. Thus, results of multiple imputations have a low risk of bias.

**Table 1.** Pooled Bivariate Pearson Correlations of Study Variables.

	1	2	3	4	5	6	M%
1. Agency	*.* *70*						24.0
2. Communion	.55**	.*74*					20.8
3. SPA ongoing development	.56**	.51**	.*87*				16.2
4. SPA physical losses	−.36**	−.31*	−.47**	.*77*			16.2
5. Self−rated physical health	.34*	.14	.27*	−.35*	.*78*		31.8
6. Self-rated emotional health	.15	.13	.20*	−.28*	.06	*.78*	31.8

*Notes. n*  =  154. All correlations are based on 20 imputed datasets and controlled for age, sex, and educational level. Internal consistencies (Cronbach's *α*) are displayed in the diagonal. M%  =  percentages of missing data per variable.

*p < .05. **p < .01.

### Relationship Between Study Variables

Table 1 displays pooled intercorrelations of study variables based on 20 imputed data sets. On a bivariate level, agency and communion were highly correlated, *r* = .55, *p* < .001, and both were significantly associated with SPA ongoing development, *r_agency_*  =  .56, *p* < .001, *r_communion_*  =  .51, *p* < .001, and SPA physical losses, *r_agency_*  =  -.36, *p* < .001, *r_communion_*  =  -.31, *p*  =  .001. However, only agency (but not communion) was found to correlate significantly with self-rated physical health, *r_agency_*  =  .34, *p*  =  .003, while correlations of agency or communion with self-reported emotional health were not significant, *p* ≥ .157.

### Associations of Agency and Communion with SPA Domains

Multiple regression analyses based on imputed data were conducted to investigate the relationship of the two SPA domains ongoing development and physical losses with agency and communion as well as self-rated physical and emotional health (see Table 2). All analyses were controlled for sex, age, and educational level, and inspection of *VIF* did not reveal collinearity (*VIF* ≤ 1.74).

**Table 2.** Regression Models for SPA Ongoing Development and SPA Physical Losses.

**Predictors**	**SPA ongoing development**	**SPA physical losses**	
	*β* **(*SE*)**	***β* (*SE*)**	***VIF***
**Agency**	0.28 (.07)**	−0.08 (.07)	1.74
**Communion**	0.23(.07)*	−0.09 (.06)	1.53
**Physical Health**	0.09 (.07)	-.0.15 (.06)*	1.19
**Emotional Health**	0.08 (.07)	−0.12 (.05)*	1.05
***R^2^***	.412	.263	

*Notes. n*  =  154. SPA  =  self-perceptions of aging; all analyses were controlled for sex, age, and educational level. VFI  =  variance inflation factor based on imputed data.

*p < .05. **p < .001.

#### SPA Ongoing Development

SPA ongoing development were significantly associated with agency, communion, self-rated physical and emotional health, *F*(4,  516.10)  =  15.24, *p* < .001. Taken together, they accounted for 41.2% of the variance (*R^2^_adjusted_*  =  .40) in SPA ongoing development. However, only agency, *β*  =  0.28, *t*(516.10)  =  3.90, *p* < .001, and communion, *β*  =  0.23, *t*(516.10)  =  3.37, *p*  =  .001, but not self-reported physical and emotional health accounted for significant unique proportions of variance in SPA ongoing development, *p* ≥ .239.

#### SPA Physical Losses

SPA physical losses were significantly associated with agency, communion, self-rated physical and emotional health, accounting for 26.3% of the variance (*R^2^_adjusted_*  =  .24) in SPA physical losses, *F*(4, 397.30)  =  7.75, *p* < .001. However, while self-rated physical, *β*  =  −0.15, *t*(397.30)  =  −2.68, *p*  =  .009, and emotional health, *β*  =  −0.12, *t*(397.30)  =  -2.35, *p*  =  .020 accounted for significant unique amounts of variance in SPA physical losses, agency and communion did not share significant unique amounts of variance with SPA physical losses, *p*  ≥ .116.

### Post-Hoc Power Analyses

Our study represents a secondary analysis of the TIGER study. Thus, sample size was not planned to be sufficiently powered for the current analyses. Since power analyses for multiple imputed data and single regression coefficients are not yet well established (Zha & Harel, 2021), we based our post-hoc power analysis on complete cases (*n*  =  80) and calculated the statistical power (*α*  =  .05) to detect an at least small effect (*d*  =  .20, Cohen (1992)) of agency, communion, physical or emotional health on SPA ongoing development or SPA physical losses. Results indicate that analyses based on complete data would have been sufficiently powered, *β* – 1  =  .80. Thus, based on simulation studies (Kontopantelis et al., 2017; Zha & Harel, 2021), it is plausible to assume that the achieved power using multiple imputations is considerably higher and that our analyses are sufficiently powered.

## Discussion

In this study, we investigated associations of agency and communion as meta-traits of personality with SPA related to ongoing development and physical losses in a sample of old-old adults in poor health. We hypothesized that higher levels of agency and communion are related to higher SPA ongoing development. Regarding SPA physical losses, we hypothesized that agency and communion are not related to this SPA domain when controlling for health. Results supported our hypotheses.

### Associations of Agency and Communion with SPA Domains

#### Results on SPA Ongoing Development

In our study, agency and communion were significantly associated with higher SPA ongoing development. This suggests that old-old adults with a stronger agentic focus on the self as well as those with a stronger communal focus on others perceive their aging more as associated with making plans and learning new things. This finding might be due to the rather general nature of development that is addressed by the items: Making many plans and putting ideas into action can refer to agentic and communal development. For instance, a person high in agency might keep up a hobby like painting even when it becomes more difficult and experience ongoing development through maintenance of or even increasing artistic capabilities. A very communal person may invest effort in contact with family and friends and gain a sense of ongoing development through focusing on social contacts.

Interestingly, physical and emotional health were only associated with SPA ongoing development on a bivariate level, but not in the regression analysis. This suggests that health does not explain unique proportions of variance beyond personality in this SPA domain, indicating that the aspect of health that is associated with SPA ongoing development is accounted for by agency and communion.

#### Results on SPA Physical Losses

On a bivariate level, agency and communion were both negatively correlated to the perception of aging-related physical losses. These associations disappeared, however, when controlling for health in the regression analysis. This finding was in line with our hypothesis and suggests that personality does not account for a unique amount of variance beyond health in SPA related to physical losses, at least in this sample of old-old adults in poor health.

Our study corroborates findings from prior studies by indicating that personality and domain-specific SPA are clearly related cross-sectionally. Past research was mostly based on the Big Five approach and yielded inconsistent results on the relationship between specific Big Five personality traits and SPA. Thus, by examining the higher-order factors of agency and communion, our study provides a more general understanding of personality and SPA, which may also be useful to further explore the relationship between the two constructs. Furthermore, our study indicates that agency and communion as fundamental dimensions of personality relate to SPA not only on the level of personal values (Blawert & Wurm, 2021), but also on the level of traits. In addition, our findings add to studies pointing to the role of agency and communion for overall adaptation (i.e., successfully and flexibly meeting situational demands) and well-being in later life by showing that these traits also relate to the way old-old adults perceive their own aging.

In late life, personality and health are closely intertwined (Mueller et al., 2018). Thus, our study helps to disentangle the effects of personality and health for SPA by showing domain-specific associations with SPA in a sample of old-old adults in poor health.

The importance of agency for goal pursuit is also stressed in the motivational theory of life-span development (e.g., Heckhausen et al., 2010). This theory posits that individuals strive to exert control over their environment to reach their goals (primary control/agency), which is increasingly impeded by accumulating health problems in old age (Heckhausen et al., 2019). Our findings on the association of agency and communion with SPA ongoing development might be a sign of successful adaptation to changing health status in old age.

### Limitations and Strengths

This study has several limitations. First, the sample is rather small and from the initial sample of 244 participants, we were only able to include data from 154 (63.11%) participants, due to drop-out and a high amount of missing data. However, data was missing completely at random, and dropouts did not differ from those who were included in our analyses in age or educational level. Second, TIGER is an intervention study, which may have affected results; however, intervention and control groups did not differ in study variables. Third, the present study was cross-sectional in design, so we cannot determine whether agency and communion affect SPA or vice versa. Furthermore, given the relatively high bivariate correlations between agency, communion and SPA, we cannot exclude the possibility that these variables are influenced by a third variable and that there is no causal relationship between them. In this regard, the investigation of self-efficacy and resilience factors as internal resources would be of interest. Self-efficacy as the perceived capability to shape one's life and environment is a central component of agency and also highly relevant in social relationships (Bandura, 2018), and higher self-efficacy is also related to more positive SPA (Tovel et al., 2019). In a similar vein, resilience as the capability to adapt successfully when faced with stressors is related to personality on the level of the Big Five traits (Oshio et al., 2018), and recent studies have found associations of resilience with SPA and attitudes towards aging (Kunuroglu & Yuzbasi, 2021; Losada-Baltar et al., 2021). A further limitation is that we cannot determine the role that the experience of hospitalization might have played in our analysis. Hospitalization could have increased the salience of health-related losses and thus could have masked a potential association of agency and communion with SPA physical losses that might emerge in another setting, survey period or population. Furthermore, we were not able to consider the type of health issue that led to hospitalization, as due to the study design, participants varied greatly in these health issues. It has to be noted that many old-old adults suffer from multiple health issues comorbidly, which makes disentangling effects of specific health issues (e.g., chronic vs. acute) difficult. In the current study, this is further complicated by the fact that chronic conditions could also have resulted in acute health events that led to hospitalization. In general, people are more likely to attribute chronic conditions to aging, while acute conditions are more likely to be attributed to illness (Stewart et al., 2012). For this reason, it would be interesting to investigate in future studies a potential moderating role of acute versus chronic health issues in the relationship of SPA with personality. Such studies should assess larger samples and include patients of a larger age range and from inpatient and outpatient settings as such samples would allow for an appropriate comparison between acute and chronic conditions.

Yet there are also several strengths to our approach. Our findings advance the understanding of the relationship between personality and SPA by first investigating the meta-traits of agency and communion. In addition, we were able to recruit an old-old sample in poor health; people in this group are usually less represented in larger studies and hence our findings give valuable insights on personality and SPA in a population in which positive SPA may be particularly threatened.

### Implications for Future Research

Our findings provide starting points for a variety of research questions. For instance, future studies should investigate the relationship between agentic and communal traits and SPA domains longitudinally to examine the nature of a potential causal relationship. It might also be of interest to investigate the role of agency and communion for SPA domains other than the ones considered in this study, e.g., SPA related to family or working life (Kornadt & Rothermund, 2012), but also subjective age or other SPA measures (for an overview of measures, see Klusmann et al. (2020) in future studies. Furthermore, these studies could investigate potential mediators or moderators of the association of agency and communion with SPA. For instance, agency and communion might pose predispositions for differential behaviors that foster the perception of ongoing development. Regarding potential moderators, investigating the role of work and family transitions, e.g., prolonged paid work or premature spousal death, but also sociodemographic factors like gender and social class indicators such as education or income might well be advantageous. Such studies should use large-scale population-based surveys to allow for valid conclusions on the general population. In addition, including physical activity as a moderator or mediator might help clarify the lack of results for SPA physical losses. It might also be of interest to investigate the role of social activities and social engagement. Moreover, agency and communion might themselves serve as mediators or moderators in the association of health and SPA (Wurm et al., 2017), given the bidirectional associations of personality and health in old and very old age (Mueller et al., 2018). It might also be interesting to investigate associations of agency and communion with SPA in healthier or younger populations to examine whether the association varies by age and/or cohort. Future studies should also investigate associations of SPA with personality traits that relate to negative outcomes, such as unmitigated agency (i.e., a focus on the self to the exclusion of others, e.g., hostility) and unmitigated communion (i.e., a focus on others to the exclusion of the self, e.g., overinvolvement with other's problems and neglect of own needs). Moreover, our findings stress the necessity of investigating multiple domains of SPA, since constructs of interest might be associated with one domain, but not with another (Boeder & Tse, 2020). Using measures of general SPA might thus mask potential associations of important factors with specific SPA domains.

## Conclusion

In this study, we advanced the understanding of SPA by showing that the “Big Two” of personality, namely agency and communion, are differentially associated with two SPA domains in a sample of old-old adults in poor health. Higher agency and communion were associated with higher SPA related to ongoing development. Conversely, neither agency nor communion were related to SPA related to physical losses when controlling for physical and emotional health. Our findings suggest that the investigation of agency and communion is effective in examining the relationship between personality traits and SPA domains. Moreover, our study showed that even in very old age and poor health, people still perceive aging-related gains in terms of ongoing development that are associated with their personality.

## References

[bibr1-00914150211050874] Abele A. E. Hauke N. (2020). Comparing the facets of the big two in global evaluation of self versus other people. European Journal of Social Psychology, 50(5), 969–982. 10.1002/ejsp.2639

[bibr2-00914150211050874] Ackerman S. Zuroff D. C. Moskowitz D. (2000). Generativity in midlife and young adults: Links to agency, communion, and subjective well-being. The International Journal of Aging and Human Development, 50(1), 17–41. 10.2190/9F51-LR6T-JHRJ-2QW6 10735180

[bibr3-00914150211050874] Austin A. Costabile K. (2017). Two routes toward optimism: How agentic and communal themes in autobiographical memories guide optimism for the future. Memory (Hove, England), 25(10), 1358–1365. 10.1080/09658211.2017.1305417 28357895

[bibr4-00914150211050874] Bakan D. (1966). The duality of human existence: An essay on psychology and religion. Rand Mcnally.

[bibr5-00914150211050874] Baltes P. B. (1987). Theoretical propositions of life-span developmental psychology: On the dynamics between growth and decline. Developmental Psychology, 23(5), 611–626. 10.1037/0012-1649.23.5.611

[bibr6-00914150211050874] Baltes P. B. Smith J. (2003). New frontiers in the future of aging: From successful aging of the young old to the dilemmas of the fourth age. Gerontology, 49(2), 123–135. 10.1159/000067946 12574672

[bibr7-00914150211050874] Bandura A. (2018). Toward a psychology of human agency: Pathways and reflections. Perspectives on Psychological Science, 13(2), 130–136. 10.1177/1745691617699280 29592657

[bibr8-00914150211050874] Bem S. L. (1974). The measurement of psychological androgyny. Journal of Consulting and Clinical Psychology, 42(2), 155–162. 10.1037/h0036215 4823550

[bibr9-00914150211050874] Bem S. L. Lewis S. A. (1975). Sex role adaptability: One consequence of psychological androgyny. Journal of Personality and Social Psychology, 31(4), 634–643. 10.1037/h0077098

[bibr10-00914150211050874] Blawert A. Wurm S. (2019). Personality in later life. In Gu D. Dupre M. E. (Eds.), Encyclopedia of gerontology and population aging (pp. 1–8). Springer International Publishing. 10.1007/978-3-319-69892-2_100-1

[bibr11-00914150211050874] Blawert A. Wurm S. (2021). Shifting self-perceptions of ageing: Differential effects of value priorities on self-perceptions of ageing beyond age stereotypes. European Journal of Ageing, 18(2), 257–267. 10.1007/s10433-020-00578-3 34220405 PMC8217379

[bibr12-00914150211050874] Boeder J. Tse D. C. (2020). Measuring self-perceptions of aging: Differences between measures when predicting health outcomes. The Journals of Gerontology: Series B, 76(5), 825–835. 10.1093/geronb/gbaa064 32392581

[bibr13-00914150211050874] Cohen J. (1992). A power primer. Psychological Bulletin, 112(1), 155. 19565683 10.1037//0033-2909.112.1.155

[bibr14-00914150211050874] Dean-Church L. Gilroy F. D. (1993). Relation of sex-role orientation to life satisfaction in a healthy elderly sample. Journal of Social Behavior and Personality, 8(1), 133–140.

[bibr15-00914150211050874] Diehl M. Owen S. K. Youngblade L. M. (2004). Agency and communion attributes in adults’ spontaneous self-representations. International Journal of Behavioral Development, 28, 1–15. 10.1080/01650250344000226 18592013 PMC2441921

[bibr16-00914150211050874] Diehl M. Wettstein M. Spuling S. M. Wurm S. (2021). Age-related change in self-perceptions of aging: Longitudinal trajectories and predictors of change. Psychology and Aging, 36(3), 344–359. 10.1037/pag0000585 33539148 PMC8096705

[bibr17-00914150211050874] Enzenbach C. Wicklein B. Wirkner K. Loeffler M. (2019). Evaluating selection bias in a population-based cohort study with low baseline participation: The LIFE-adult-study. BMC Medical Research Methodology, 19(1), 135. 10.1186/s12874-019-0779-8 31262266 PMC6604357

[bibr18-00914150211050874] Faul F. Erdfelder E. Buchner A. Lang A.-G. (2009). Statistical power analyses using G* power 3.1: Tests for correlation and regression analyses. Behavior Research Methods, 41(4), 1149–1160. 10.3758/BRM.41.4.1149 19897823

[bibr19-00914150211050874] Heckhausen J. Wrosch C. Schulz R. (2010). A motivational theory of life-span development. Psychological Review, 117(1), 32–60. 10.1037/a0017668 20063963 PMC2820305

[bibr20-00914150211050874] Heckhausen J. Wrosch C. Schulz R. (2019). Agency and motivation in adulthood and old age. Annual Review of Psychology, 70(1), 191–217. 10.1146/annurev-psych-010418-103043 30110574

[bibr21-00914150211050874] Helgeson V. S. (1994). Relation of agency and communion to well-being: Evidence and potential explanations. Psychological Bulletin, 116(3), 412–428. 10.1037/0033-2909.116.3.412

[bibr22-00914150211050874] Kandler C. Zimmermann J. McAdams D. P. (2014). Core and surface characteristics for the description and theory of personality differences and development. European Journal of Personality, 28(3), 231–243. 10.1002/per.1952

[bibr23-00914150211050874] Kleinspehn-Ammerlahn A. Kotter-Grühn D. Smith J. (2008). Self-perceptions of aging: Do subjective age and satisfaction with aging change during old age? The Journals of Gerontology Series B: Psychological Sciences Social Sciences, 63(6), 377–385. 10.1093/geronb/63.6.P377 19092041

[bibr24-00914150211050874] Klusmann V. Notthoff N. Beyer A.-K. Blawert A. Gabrian M. (2020). The assessment of views on ageing: A review of self-report measures and innovative extensions. European Journal of Ageing, 17(4), 403–433. 10.1007/s10433-020-00556-9 33376461 PMC7752934

[bibr25-00914150211050874] Kontopantelis E. White I. R. Sperrin M. Buchan I. (2017). Outcome-sensitive multiple imputation: A simulation study. BMC medical Research Methodology, 17(1), 1–13. 10.1186/s12874-016-0281-5 28068910 PMC5220613

[bibr26-00914150211050874] Kornadt A. Siebert J. Wahl H.-W. (2019). The interplay of personality and attitudes toward own aging across two decades of later life. PloS one, 14, e0223622. 10.1371/journal.pone.0223622 31596876 PMC6785129

[bibr27-00914150211050874] Kornadt A. E. Kessler E.-M. Wurm S. Bowen C. E. Gabrian M. Klusmann V. (2020). Views on ageing: A lifespan perspective. European Journal of Ageing, 17(4), 387–401. 10.1007/s10433-019-00535-9 33380996 PMC7752932

[bibr28-00914150211050874] Kornadt A. E. Rothermund K. (2012). Internalization of age stereotypes into the self-concept via future self-views: A general model and domain-specific differences. Psychology and Aging, 27(1), 164–172. 10.1037/a0025110 21875214

[bibr29-00914150211050874] Kunuroglu F. Yuzbasi D. V. (2021). Factors promoting successful aging in turkish older adults: Self compassion, psychological resilience, and attitudes towards aging. Journal of Happiness Studies, 1–16. 10.1007/s10902-021-00388-z

[bibr30-00914150211050874] Laidlaw K. Power M. J. Schmidt S. Group W.-O. (2007). The attitudes to ageing questionnaire (AAQ): Development and psychometric properties. International Journal of Geriatric Psychiatry, 22(4), 367–379. 10.1002/gps.1683 17051535

[bibr31-00914150211050874] Lawton M. P. (1975). The Philadelphia geriatric center morale scale: A revision. Journal of Gerontology, 30(1), 85–89. 1109399 10.1093/geronj/30.1.85

[bibr32-00914150211050874] Lefkowitz E. S. Zeldow P. B. (2006). Masculinity and femininity predict optimal mental health: A belated test of the androgyny hypothesis. Journal of Personality Assessment, 87(1), 95–101. 10.1207/s15327752jpa8701_08 16856790

[bibr33-00914150211050874] Levy B. R. (2003). Mind matters: Cognitive and physical effects of aging self-stereotypes. The Journals of Gerontology Series B: Psychological Sciences and Social Sciences, 58(4), 203–211. 10.1093/geronb/58.4.P203 12878645

[bibr34-00914150211050874] Loi S. M. Dow B. Moore K. Hill K. Russell M. Cyarto E. Lautenschlager N. T. (2015). Attitudes to aging in older carers–do they have a role in their well-being? International psychogeriatrics, 27(11), 1893–1901. 10.1017/S1041610215000873 26073317

[bibr35-00914150211050874] Losada-Baltar A. Jiménez-Gonzalo L. Gallego-Alberto L. Pedroso-Chaparro M. d. S. Fernandes-Pires J. Márquez-González M. (2021). We are staying at home.” association of self-perceptions of aging, personal and family resources, and loneliness with psychological distress during the lock-down period of COVID-19. The Journals of Gerontology: Series B, 76(2), e10–e16. 10.1093/geronb/gbaa048 PMC7184373 32282920

[bibr36-00914150211050874] Mansfield E. D. McAdams D. P. (1996). Generativity and themes of agency and communion in adult autobiography. Personality and Social Psychology Bulletin, 22(7), 721–731. 10.1177/0146167296227006

[bibr37-00914150211050874] Martin A. E. Slepian M. L. (2020). Big Two, The. In Zeigler-Hill V. Shackelford T. K. (Eds.), Encyclopedia of personality and individual differences (pp. 472–474). Springer International Publishing. 10.1007/978-3-319-24612-3_864

[bibr38-00914150211050874] Matud M. P. Bethencourth J. M. Ibáñez I. Fortes D. (2020). Gender and psychological well-being in older adults. International psychogeriatrics, 32(11), 1293–1302. 10.1017/S1041610220000824 32456745

[bibr39-00914150211050874] McAdams D. P. de St Aubin E. (1992). A theory of generativity and its assessment through self-report, behavioral acts, and narrative themes in autobiography. Journal of Personality and Social Psychology, 62(6), 1003–1015. 10.1037/0022-3514.62.6.1003

[bibr40-00914150211050874] Moor C. Zimprich D. Schmitt M. Kliegel M. (2006). Personality, aging self-perceptions, and subjective health: A mediation model. The International Journal of Aging and Human Development, 63(3), 241–257. 10.2190/AKRY-UM4K-PB1V-PBHF 17152411

[bibr41-00914150211050874] Mueller S. Wagner J. Smith J. Voelkle M. C. Gerstorf D. (2018). The interplay of personality and functional health in old and very old age: Dynamic within-person interrelations across up to 13 years. Journal of Personality and Social Psychology, 115(6), 1127–1147. 10.1037/pspp0000173 29189025

[bibr42-00914150211050874] O’brien R. M. (2007). A caution regarding rules of thumb for variance inflation factors. Quality & Quantity, 41(5), 673–690. 10.1007/s11135-006-9018-6

[bibr43-00914150211050874] O'Shea D. M. Dotson V. M. Fieo R. A. (2017). Aging perceptions and self-efficacy mediate the association between personality traits and depressive symptoms in older adults. International Journal of Geriatric Psychiatry, 32, 1217–1225. 10.1002/gps.4584 27653811

[bibr44-00914150211050874] Oshio A. Taku K. Hirano M. Saeed G. (2018). Resilience and Big five personality traits: A meta-analysis. Personality and Individual Differences, 127, 54–60. 10.1016/j.paid.2018.01.048

[bibr45-00914150211050874] Paulhus D. L. Trapnell P. D. (2008). Self-presentation of personality. In John O. P. Robins R. W. Pervin L. A. (Eds.), Handbook of personality psychology (Vol. 19 (pp. 492–517). Guilford.

[bibr46-00914150211050874] Perrig-Chiello P. Hutchison S. (2010). Health and well-being in Old Age: The pertinence of a gender mainstreaming approach in research. Gerontology, 56(2), 208–213. 10.1159/000235813 19707008

[bibr47-00914150211050874] R Core Team. (2019). *R: A language and environment for statistical computing*. https://www.R-project.org/

[bibr48-00914150211050874] Rimmele M. Wirth J. Britting S. Gehr T. Hermann M. van den Heuvel D. Volkert D. (2021). Improvement of transitional care from hospital to home for older patients, the TIGER study: Protocol of a randomised controlled trial. BMJ open, 11(2), e037999. https://doi:10.1136/bmjopen-2020-037999 10.1136/bmjopen-2020-037999 PMC7871673 33558344

[bibr49-00914150211050874] Robitzsch A. Grund S. (2021). miceadds: Some Additional Multiple Imputation Functions, Especially for ‘mice’. *R package version 3.11-6.* https://CRAN.R-project.org/package = miceadds.

[bibr50-00914150211050874] Rosseel Y. (2012). Lavaan: An R package for structural equation modeling. Journal of Statistical Software, 48(2), 1–36. http://www.jstatsoft.org/v48/i02/

[bibr51-00914150211050874] Runge T. E. Frey D. Gollwitzer P. M. Helmreich R. L. Spence J. T. (1981). Masculine (instrumental) and feminine (expressive) traits. Journal of Cross-Cultural Psychology, 12(2), 142–162. 10.1177/0022022181122002

[bibr52-00914150211050874] Rupprecht F. S. Dutt A. J. Wahl H.-W. Diehl M. K. (2019). The role of personality in becoming aware of Age-related changes. GeroPsych, 32(2), 57–67. 10.1024/1662-9647/a000204 32362819 PMC7194201

[bibr53-00914150211050874] Sarkisian C. A. Hays R. D. Berry S. Mangione C. M. (2002). Development, reliability, and validity of the expectations regarding aging (ERA-38) survey. The Gerontologist, 42(4), 534–542. 10.1093/geront/42.4.534 12145381

[bibr54-00914150211050874] Spence J. T. Helmreich R. L. Stapp J. (1974). The personal attributes questionnaire: A measure of sex role stereotypes and masculinity-femininity. University of Texas.

[bibr55-00914150211050874] Stephan Y. Demulier V. Terracciano A. (2012). Personality, self-rated health, and subjective age in a life-span sample: The moderating role of chronological age. Psychology and Aging, 27(4), 875–880. 10.1037/a0028301 22582885 PMC3439601

[bibr56-00914150211050874] Steverink N. Westerhof G. J. Bode C. Dittmann-Kohli F. (2001). The personal experience of aging, individual resources, and subjective well-being. The Journals of Gerontology Series B: Psychological Sciences and Social Sciences, 56(6), 364–373. 10.1093/geronb/56.6.P364 11682590

[bibr57-00914150211050874] Stewart T. L. Chipperfield J. G. Perry R. P. Weiner B. (2012). Attributing illness to ‘old age:’consequences of a self-directed stereotype for health and mortality. Psychology & Health, 27(8), 881–897. 10.1080/08870446.2011.630735 22149693

[bibr58-00914150211050874] Tovel H. Carmel S. Raveis V. H. (2019). Relationships Among self-perception of aging. Physical Functioning, and Self-efficacy in Late Life. The Journals of Gerontology: Series B, 74(2), 212–221. 10.1093/geronb/gbx056 28549180

[bibr59-00914150211050874] Turner S. G. Hooker K. (2020). Are thoughts about the future associated With perceptions in the present?: Optimism. Possible Selves, and Self-Perceptions of Aging. The International Journal of Aging and Human Development. 10.1177/0091415020981883 33369480

[bibr60-00914150211050874] UNESCO (1997). ISCED 1997: International standard classification of education.

[bibr61-00914150211050874] Vafaei A. Ahmed T. Freire A. d. N. F. Zunzunegui M. V. Guerra R. O. (2016). Depression, Sex and gender roles in older adult populations: The international mobility in aging study (IMIAS). PloS one, 11(1), e0146867. 10.1371/journal.pone.0146867 26771828 PMC4714885

[bibr62-00914150211050874] Van Buuren S. (2018). Flexible imputation of missing data. CRC press.

[bibr63-00914150211050874] van Buuren S. Groothuis-Oudshoorn K. (2011). mice: Multivariate Imputation by Chained Equations in R. *2011, 45*(3), 67. 10.18637/jss.v045.i03

[bibr64-00914150211050874] Vecchione M. Alessandri G. Barbaranelli C. Caprara G. (2011). Higher-order factors of the big five and basic values: Empirical and theoretical relations. British Journal of Psychology, 102(3), 478–498. https://doi:10.1111/j.2044-8295.2010.02006.x 21752001 10.1111/j.2044-8295.2010.02006.x

[bibr65-00914150211050874] Ware J. E. J. Kosinski M. Keller S. D. (1996). A 12-item short-form health survey: Construction of scales and preliminary tests of reliability and validity. Medical Care, 220–233. https://doi:10.1097/00005650-199603000-00003 8628042 10.1097/00005650-199603000-00003

[bibr66-00914150211050874] Welzel C. Inglehart R. (2010). Agency, values, and well-being: A human development model. Social Indicators Research, 97(1), 43–63. https://doi:10.1007/s11205-009-9557-z 20390031 10.1007/s11205-009-9557-z PMC2848347

[bibr67-00914150211050874] Westerhof G. J. Miche M. Brothers A. F. Barrett A. E. Diehl M. Montepare J. M. Wurm S. (2014). The influence of subjective aging on health and longevity: A meta-analysis of longitudinal data. Psychology and Aging, 29(4), 793–802. 10.1037/a0038016 25365689

[bibr68-00914150211050874] Wirtz M. A. Morfeld M. Glaesmer H. Brähler E. (2018). Normierung des SF-12 version 2.0 zur messung der gesundheitsbezogenen lebensqualität in einer deutschen bevölkerungsrepräsentativen stichprobe. Diagnostica, 64, 215–226. 10.1026/0012-1924/a000205

[bibr69-00914150211050874] Wolff J. K. Warner L. M. Ziegelmann J. P. Wurm S. (2014). What do targeting positive views on ageing add to a physical activity intervention in older adults? Results from a randomised controlled trial. Journal of Psychology and Health, 29(8), 915–932. https://doi:10.1080/08870446.2014.896464 24559210 10.1080/08870446.2014.896464

[bibr70-00914150211050874] Wurm S. Diehl M. Kornadt A. E. Westerhof G. J. Wahl H.-W. (2017). How do views on aging affect health outcomes in adulthood and late life? Explanations for an established connection. Developmental Review, 46, 27–43. 10.1016/j.dr.2017.08.002 33927468 PMC8081396

[bibr71-00914150211050874] Wurm S. Tesch-Römer C. Tomasik M. (2007). Longitudinal findings on aging-related cognitions, control beliefs, and health in later life. The Journals of Gerontology Series B: Psychological Sciences and Social Sciences, 62(3), 156–164. 10.1093/geronb/62.3.P156 17507583

[bibr72-00914150211050874] Wurm S. Wiest M. Wolff J. K. Beyer A. K. Spuling S. M. (2020). Changes in views on aging in later adulthood: The role of cardiovascular events. European Journal of Ageing, 17(4), 457–467. 10.1007/s10433-019-00547-5 33380999 PMC7752931

[bibr73-00914150211050874] Zha R. Harel O. (2021). Power calculation in multiply imputed data. Statistical Papers, 62(1), 533–559. 10.1007/s00362-019-01098-8

[bibr74-00914150211050874] Zhang Z. (2016). Multiple imputation with multivariate imputation by chained equation (MICE) package. Annals of Translational Medicine, 4(2), 30. 10.3978/j.issn.2305-5839.2015.12.63 26889483 PMC4731595

